# Progress in Medicine

**Published:** 1894-12-29

**Authors:** 


### Progress in Medicine.

INFECTIVE DISEASES—(*continued from page* 155).

**Typhoid.**—As to treatment, Von Jaksch[1] reports favourably of lactophenin in doses of ½ to 1 grain, repeated several times in a day, according to its anti-pyretic and sedative effects. Tricresol, which is five or six times more powerful than carbolic acid, yet less poisonous, and not easily absorbed when dissolved in olive oil and soft soap, has been given in capsules by Kölsch,[2] with good results. Three capsules, each containing 1½ grains, increased gradually to twelve daily, were given, and rapid progress was observed. McNutt[3] recommends perchloride of iron every two or four hours for its effect on the mucous membrane and general condition. He adds sometimes liq. hydrarg. or Fowler's solution. His results are confirmed by Wedgwood, who treated twenty-one cases with success and with amelioration of all symptoms.[4] Fisk recommends[5] an initial dose of calomel, followed by turpentine, bismuth, and castor oil in small repeated doses. Kraus and Buswell point out that in typhoid we have to combat both the infection and the intoxication from the toxalbumins. By injection of a bouillon culture of B. pyocyaneus sterilised by heat the natural resistance of the cells is greatly increased, and in animals so treated typhoid inoculation failed, while the controls died.[6]

Broadbent[7] emphasizes the need of constantly watching the stools during the progress of the disease and modifying the diet accordingly. Stimulants should be used chiefly in later stages when a frequent weak pulse, dry tongue, and mental confusion are present. From one ounce of brandy to a maximum of ten in a day may be given. For sleeplessness cold sponging or opium and occasionally phenacetin are to be preferred. Though nothing has been found capable of destroying the bacilli in the blood or neutralising their products, he gives drachm doses of liq. hydrarg. every three or four hours for one or two days, when offensive watery diarrhœa occurs, to remove from the bowel the causes of secondary infection. The effect on the accompanying nervous symptoms is often striking. A class of cases marked by early repeated vomiting due to a ptomaines is often relieved by mercury. Strong beef-tea and undigested milk are the cause of most other instances of diarrhœa. Tympanites from local lesions may be treated by iced compresses or hot ones, but when accompanied by great nervous prostration and retention of urine, large doses of opium, two grains or more, are invaluable. Severe initial headache or enlargement of the liver are often precursors of perforation and hæmorrhage later on. For the latter he gives full doses of opium and ergotin, and employs the ice-bag. He notices that antipyrin prevents the elimination of toxins (Roque, however, thinks it actually prevents their formation) while cold baths increase this. For the reduction of high temperatures cold baths are best, and the continuous bath system of Dr. Barr, where the water is kept at 95 deg., or lower, appears to reduce the mortality to only 2 or 3 per cent., and deserves to be popular. Daily tepid sponging in any case is desirable, and wet cloths on the abdomen are often beneficial. In collapse ether and strychnia in addition to brandy may be needed. The paper is a most instructive one, and with the reports next to be considered forms, perhaps, the most important contribution of the year.

From Johns Hopkins' University reports[8] we find that the patients there have a bath of 65 deg. to 70 deg. every third hour when the rectal temperature is at least 102·5 deg. The mortality is only 7·1 per cent., or about half the usual rate in America. Cold affusions to the head and friction of the limbs are employed during the bath. The tonic effect of the bath was much greater than the antipyretic. Hæmorrhage and relapse were rather more frequent than usual. Six of the eight reports are contributed by Dr. Osler, who treated 229 cases, to 196 of which this method was used. In discussing the anæmia of the fever the absence of leucocytosis, with a relative increase of mononuclear leucocytes is noted as peculiar to the disease.

There were twenty-one cases of definite nephritis. The kidney lesions were such as might occur in any acute disease, and were produced by the bacteria or their products. The diazo reaction which was noted in 136 of the 196 cases is of diagnostic value in typhoid when found in concurrence with other symptoms. However, it disappears in the second week, and is common in tuberculosis, pneumonia, measles, and scarlatina. We may here remark that Dawson[9] also finds it more frequently in typhoid than in any other disease except perhaps measles and phthisis, thus its absence is a strong argument against typhoid in the early stages.

In the Bristol discussion[10] on pyrexia Hale White elaborated his views as to its nervous causation. In hyperpyrexia the cold bath is the best treatment. Simple pyrexia should not be interfered with, but in typhoid, if the temperature is over 102·5, cold baths cause a reduction in the death rate, not only by reducing the heat, but also increasing enormously the elimination of toxins by the urine. Antipyretic drugs are rarely desirable. Lindsay recommended for hyperpyrexia the cold bath and 20 or 30 grain doses of quinine. Skerritt was in favour of 30 or 40 grains of quinine. Its effect lasts longer than that of other drugs. Barr also warns against treating simple pyrexia. In severe typhoid he keeps the patient in a permanent bath. Evaporating poultices and iced-air treatment are also of value. D. Powell remarked that pyrexia inhibited the activity of microbes. Garrett Anderson agreed that the importance of temperature depended on its cause. In hysteria and relapsing fever it was unimportant. Shingleton Smith thought that phenacetin might replace the bath in moderate cases. Pope found cold baths reduced the mortality. Grainger Stewart in milder cases preferred cold sponging, ice packs, and antipyretics; and Osler replied that though cold baths were disagreeable and troublesome, he felt bound to adopt them, as they saved three or four lives in the hundred.

Special apparatus for lifting patients in and out of baths is described by Bellamy,[11] but Noyes[12] points out that the bath can be given without moving the patient, by laying under him a large rubber sheet, raising the edges by thick sandbags, clamping the angles, and then filling it with water. The fluid can be removed by a syphon, and the sheet taken away with very little trouble. Springthorpe, from Australia,[13] would substitute for milk diet a hopped malt extract previously sterilised. Forty cases were fed upon it with entire absence of tympanites. The deficiencies of a milk diet are thus avoided. Milk food is interfered with by acid medicines, and often causes hard curds to pass along the bowel. Certainly, in theory, his malt extract diet has very many advantages. Lesser,[14] Page, Licorish, and others actually advocate the giving no food but water, for a week or more, at a time when the stomach is irritable. In hæmorrhage the icebag and coated pills of subsulphate of iron are recommended by the *Therapeutic Gazette*. If heart failure follows, transfusion and bandaging the extremities is preferable to the use of stimulants, as less likely to increase the bleeding.

Persistent arhythmia of the pulse occurring regularly is seen in most fatal cases, while complete disappearance of the first sound is very rare, according to Hayem. Galliard notices the recovery of a case where the first sound at the base was absent.[15] This Picot considers to be a sign of myocarditis, but Huchard points out that many cardiac complications are too transient to be ascribed to organic lesions, and often depend on functional vagus disturbances caused by toxins.[16] Two cases under the care of the late Dr. Sturges, showing the difficulty of diagnosis from pure meningitis, are reported. One had general hyperæsthesia, headache and spinal pain, tremors, and attacks of head nodding; the other had violent delirium, rigidity of limbs, hyperæsthesia, and retraction of abdomen, but hæmorrhage and the progress of the disease cleared up the doubts.[17] Two cases of fœcal impaction where no tumour could be felt, and no constipation or distension had occurred, ended fatally, one by sudden collapse with pain, and the other without either symptom.[18] Dr. Cribb does not suggest any means of diagnosis in such cases. Recurrence of well-marked typhoid nine months after the first attack is recorded by Hand and Patek[19]; a case of cancrum oris[20] by Bewly; phlegmasia alba, where a clot in the left crural vein contained typhoid bacilli, by Hanshalter[21]; gangrene of both legs with amputation by Durand[22]; and double suppurative parotitis by Gilbert.[23] The last case developed later on an attack of biliary colic. This is rare, but other instances of infection of the biliary tract by Eberth's bacillus have been recorded. Goodall has had a case of typhoid where death followed the first symptom of perforation in ten minutes, and where the opening was found to be three feet and a half above the valve; both circumstances are, of course, most remarkable for their rarity.[24] Perforation has been undoubtedly survived in a few instances, and Burton Fanning reports a case where the temperature fell 10 degrees from 105, and was followed by collapse, vomiting, distension, and hiccough, yet recovery took place.[25] It would seem to have been an instance of perforation. In the same patient a perforation of the soft palate occurred later on. Laryngeal ulcers have long been observed in rare instances, but Watson Williams has for the first time demonstrated the presence in them of the typhoid bacillus.[26] His case was of a virulent type, and there was strong evidence of the disease being communicated by the breath or expectoration. The proof that the bacillus actually existed in the larynx gives, therefore, strong support to this view, and shows the great care needed in typhoid nursing. Buschke reviews the literature of bacilli found in osteo-myelitis, and details a case in which, seven years after typhoid and the appearance of a painful lump on a rib, the bacilli were found in the pus from the excised tumour.[27] Klemm, in another paper, discusses the bone lesions produced, also showing that the bacilli long remain virulent.[28]

The official report on the epidemic at Worthing 1893, shows the contamination of the water supply, possibly through a fissure in the chalk, which conveyed sewage matter into a new well. This had been sunk shortly before the outbreak to obtain an additional supply, and quickly converted the town into a hotbed of typhoid, 1,411 cases occurring, though probably some additional sources of contagion existed.[29] Professor Kelly does not think that faulty sewers or the chalk fissure were responsible,[30] but that the water was infected by some of the labourers employed on the works. Klein found both the B. coli and typhosus in the water from a main. However, the town has provided a new water supply at great expense. A serious milk epidemic is reported from New Jersey,[31] where 107 persons were attacked. The *Lancet* correspondent makes the statement that typhoid is almost unknown among the native Egyptians, though it is common enough among immigrants.[32] However, only six cases occurred among the 6,000 European visitors at Cairo last winter, newspaper reports being much exaggerated. The *Pioneer Mail* suggests to the Indian Government a trial of the filters which have proved so successful in the French army, as a means of reducing the frightful mortality from typhoid among English troops in India.[33]

Sir C. A. Cameron's address at Bristol was of high interest.[34] He pointed out the inadequacy of a purely chemical examination of water. It fails to detect the presence of small amounts of specific pollution. All it can tell is that the greater the quantity of organic nitrogen is, the greater is the probability of specific infection. The evidence as to the dangers of sewer air is most contradictory. Bristol, which is singular in having unventilated sewers, has, however, a very low typhoid rate. Still he believes that the greater number of typhoid cases arise from an air infection rather than from milk or water. The health of towns is enormously decreased by pollution of the soil, and the typhoid bacillus can live for a considerable time in fairly dry soils, but for much less in a water-logged clay. The low-lying gravelly parts of Dublin happen to have a dry subsoil and typhoid is prevalent; while in the clay soil parts the ground water is high and the typhoid germs die out. Dublin has a much better water supply than London, but the soil is more polluted by leaky drains and typhoid is far more prevalent. He notes the slight effect carbolic has in destroying typhoid bacilli, and advises the addition of an equal volume of a 5 per cent. solution of sulphuric acid with a little Condy to typhoid stools for two hours before they are poured away.

Sanerelli,[35] in a lengthy paper in the *Annals de l'Institut Pasteur*, concludes that the typhoid bacillus acts by means of a toxin which affects the nerve centres and the mucous membranes. In human as in experimental typhoid there are no bacilli (see below) in the intestinal contents, but the lesions are present. They are produced by the toxins, while the germs work as usual only in the lymphatic gland tissue. The injury done to the intestinal wall by the toxin favours the growth of B. coli, which is responsible for the ulceration. Typhoid spots contain no bacilli, and they can be produced in monkeys by injecting the toxin alone. Typhoid antitoxin destroys also the B. coli.

Klein[36] combats the view of the identity of the B. typhosus and B. coli: (*a*) The typhoid one is distinctly more cylindrical, and forms in cultures cylinders and filaments; (*b*) its motility is greater; (*c*) it grows less quickly on and in gelatine; (*d*) it forms a peculiar iridescence on the surface of gelatine; (*e*) it does not form gas bubbles in gelatine; (*f*) it does not curdle milk; (*g*) it does not form indol in broth; (*h*) it does not make gelatine uniformly turbid in twenty-four hours, differing in all these points from B. coli. He entirely denies Sanerelli's statement that typhoid bacilli are not found in the discharges. It can easily be isolated from them, at least after the first week of the disease.

The characters of the two organisms remain, moreover, fixed during successive cultivations, and they can both be isolated in many polluted waters, but only when large quantities are employed. He does not deny the possibility of transitional forms, but none such have at present been found. The organism found in water by Cameron and M'Weeney,[37] which was supposed to have caused typhoid, was clearly a form of B. Coli, and certainly not allied to B. typhosus. Moreover, only ten drops of the water were used for the cultures.

Now, it is argued that the two germs produce similar results when injected into the peritoneum, but many others do just the same, and Klein maintains that the B. Coli and typhosus thus passed through animals always remain distinct. So, too, when cultivated in sewage, but the B. typhosus soon dies out there, as he has ascertained by numerous trials.

The *Lancet*, commenting on a case of Dr. Webber's,[38] would refer the effect of sewer gas, not to the direct action of germs, of which very few are present in it, but to a toxin or gaseous ptomaine. This lowers the resistance of the system, causing marked anæmia, &c., though not directly producing any specific disease. Thus Alessi found that animals exposed to sewer-gas fell victims to specific inoculations, which were not fatal to others living out of its reach. They, however became less susceptible as they grew accustomed to the exhalations. At New York, A. C. Abbott[39] denied that the air of sewers is under a great tension, or differs much chemically from other air, or that sewer bubbles are of account by their setting free bacteria in the air.

Jacobi, in the same discussion, held the view that only irritating substances and not organisms could rise from sewers, and noticed that a thousand cases of pseudo-diphtheria, mapped out lately in New York, showed no connection with leaky sewers. At Buda Pesth Draschke, speaking on the water-borne theory of typhoid,[40] showed by elaborate statistics that improved water supplies, and not otherwise improved sanitary conditions, were the chief instruments in lowering the typhoid mortality. This was seen in the effect of fresh water supplies in Munich and Vienna. In the latter city the temporary reintroduction of Danube water was followed by a rise of typhoid mortality in the parts where it was used many times greater than that in parts where the Hochquellen was supplied, Similar evidence was supplied from Naples, Bremen. Buda, Lemberg, and Fünfkirchen.

Chantemesse[41] remarked that the water theory was commonly accepted, and denied that there was any proof of the transformation of B. Coli into B. typhosus. Yellow fever had left Vera Cruz since spring water was brought to the town, tubercle bacilli could remain alive for 70 days in sterilised water, and Laveran believed in the conveyance of malaria by drinking water. Many other diseases, not often so regarded, are at times water-borne.

Thresh[42] holds that chemical analyses, apart from a knowledge of the source of the nitrites, chlorides, and other elements, cannot guarantee water as free from risk, though it may point out impurities. Even bacteriological examination may fail from the fewness of the microbes, or when the pollution is only occasional. Lane Notter[43] reported some experiments on the Chamberland and Berkefeld filters, showing that all waters were rendered sterile by them. The introduction of the former in the French army was followed by the reduction of typhoid mortality by one half. Stoddart said that with quite new filters, or again, after their use for some weeks, the filtered water was not completely sterile ; and in a latter paper[44] he concludes as the result of his experiments, and from recent facts, that though perfctly carried out filtration is of value, it is no absolute protection. Dilution is insufficient because the mixture of sewage matter may be occasional. Typhoid germs may adhere to and settle on stones and be shaken off months after they have disappeared from the water, and the sterilizing action of light fails in slightly turbid water at a point nearer to the surface than that at which a white paper ceases to be visible. On the whole, he regards chemical analysis as the best guide we have to the value of a water ; but if this shows the presence of sewage products, or if we know otherwise of their introduction, the water is dangerous and cannot be rendered safe.

[1] Med. Rec., Sept. 8. [2] Med. Week. July 13. [3] Int. Med. Mag., June. [4] B.M.J., May 26. [5] Med. Week, June 22. [6] B.M.J., Aug. 11. [7] Lancet, Aug. 25. [8] Johns Hopkins Rep., June. [9] Am. J. Med. Sc , April. [10] B.M.J., Nov. 17. [11] Med. Rec., Aug. 25. [12] Med. Rec., July 17. [13] N. York M. J., Sept. 15. [14] Med. Rec., June 2 and May 26. [15] Med. Week, June 22. [16] Med. Week, Aug. 3. [17] Lancet, Aug. 8. [18] Int. Med., May 18. [19] Med. News, April. [20] Practit., June. [21] Dublin J. Med. Sc., June. [22] Therap. Gaz., Sept. 15. [23] Med. Week, July 27. [24] Lancet, June 23. [25] Lancet, June 30. [26] J. Laryngol., Oct. [27] Fortsch d. Medic., Aug. [28] Arch. f. Klin. Med., April. [29] B.M.J., July 21. [30] Lancet, Sept. 22. [31] Med. Rec., June 9. [32] Lancet, June 23. [33] Lancet, June 2. [34] Dublin J. Med. Sc., Aug. [35] Int. Med. Mag., July. [36] B.M.J., Oct. 13. [37] Dublin J. Med. Sc., Aug. [38] Lancet, Sept. 29. [39] Med. Rec., June 2. [40] Lancet, Sept. 15. [41] B.M.J., Sept. 22. [42] B.M.J., Aug. 18. [43] B.M.J., Aug. 18. [44] Public Health, Aug.

